# Human Bocavirus Infection in Children with Gastroenteritis, Brazil

**DOI:** 10.3201/eid1311.060671

**Published:** 2007-11

**Authors:** Maria Carolina M. Albuquerque, Ludmila N. Rocha, Fabrício José Benati, Caroline C. Soares, Adriana G. Maranhão, Maria Liz Ramírez, Dean Erdman, Norma Santos

**Affiliations:** * Universidade Federal do Rio de Janeiro, Rio de Janeiro, Brazil; † Centers for Disease Control and Prevention, Atlanta, Georgia, USA

**Keywords:** Human bocavirus, viral gastroenteritis, viral diagnostics, children, Brazil, dispatch

## Abstract

Human bocavirus (HBoV) was detected in 14 (2%) of 705 fecal specimens from Brazilian children with gastroenteritis. Coinfection with rotavirus, adenovirus, or norovirus was found in 3 (21.4%) HBoV-positive specimens. None of the HBoV-positive patients had respiratory symptoms.

Human bocavirus (HBoV) was first identified in pooled human respiratory tract specimens from Swedish children in 2005 and was provisionally classified within the genus *Bocavirus* of the family *Parvoviridae* (*1*). Previously, the only parvovirus known to be pathogenic in humans was B19 virus, which is responsible for Fifth disease in children (*2*). Because HBoV was first found in respiratory specimens, most epidemiologic studies have focused on such specimens. Shortly after its first description in Sweden, HBoV was detected in respiratory tract specimens from patients with respiratory illness in several parts of the world (*3**–**8*).

Other members of the family *Parvoviridae* that infect animals cause diseases such as leukopenia/enteritis syndrome, seen most commonly in dogs 8–12 weeks of age, with clinical features of vomiting, anorexia, lethargy, and diarrhea that lead to rapid dehydration (*9*). For this reason, we hypothesized that HBoV may play a role in human gastrointestinal disease. In this study, we retrospectively tested stool specimens collected from 2003 through 2005 from Brazilian children with acute diarrhea to investigate whether this virus can infect the human gastrointestinal tract and be detected in feces, and to assess the frequency of such infections.

## The Study

A total of 705 stool specimens from Brazilian children <15 years of age (median 3.5 years) with acute diarrhea were obtained from January 2003 through December 2005 and screened by PCR for HBoV DNA. Of these specimens, 285 (40.4%) were collected from hospitalized patients and 420 (59.6%) from outpatients: 142 (20.2%) from the emergency department and 278 (39.4%) from walk-in clinics. Only 1 specimen was obtained per patient. A total of 314 (44.5%) patients were <2 years of age, 190 (27%) were 2–5 years of age, 120 (17%) were 6–10 years of age, and 61 (8.6%) were 11–15 years of age. Age was not known for 21 patients. Relevant clinical information was collected on a standard questionnaire. This information included hospitalization status, age, sex, and clinical symptoms.

Specimens were collected at university hospitals in 3 different cities in Brazil located in areas with distinct sanitation conditions and socioeconomic backgrounds. The specimens were previously tested for other enteric viruses and bacteria, including rotavirus, norovirus, astrovirus, adenovirus, *Escherichia coli*, *Salmonella* spp., *Yersinia enterocolitica*, *Campylobacter* spp., and *Shigella* spp. (*10**,**11*). The study protocol was reviewed and approved by the Ethics Committee of the Instituto de Puericultura e Pediatria Martagão Gesteira of the Federal University of Rio de Janeiro.

Stool suspensions were prepared as 10% (w/v) in phosphate-buffered saline (pH 7.2), clarified by centrifugation at 2,500× *g* for 5 min. Two hundred microliters of each suspension was used for DNA extraction with the Wizard Genomic DNA Purification Kit (Promega, Madison, WI, USA) according to the manufacturer’s instructions. PCRs were performed as described (*7*) by using forward primer HBoV 01.2 (5′-TATGGCCAAGGCAATCGTCCAAG-3′) and reverse primer HBoV 02.2 (5′-GCCGCGTGAACATGAGAAACAGA-3′) for the nonstructural protein 1 gene. A 291-bp amplicon was generated. DNA samples were subjected to 1 cycle at 95°C for 15 min, followed by 45 cycles at 94°C for 20 s, 56°C for 20 s, and 72°C for 30 s, and a final extension at 72°C for 5 min. PCR products were detected by agarose gel electrophoresis and staining with ethidium bromide.

To confirm the presence of HBoV, amplified DNAs of PCR-positive samples were purified by using the Wizard SV gel and PCR Clean-Up system kit (Promega). Sequences were determined by using the BigDye Terminator Cycle Sequencing Kit and the ABI PRISM 3100 automated DNA sequencer (Applied Biosystems, Foster City, CA, USA). DNA sequences were assembled and analyzed with the SeqMan, EditSeq, and MegAlign programs in the Lasegene software package (DNASTAR, Madison, WI, USA). Nucleotide sequences obtained in this study were deposited in GenBank under accession nos. EF560205–EF560216.

Fourteen (2%) of 705 diarrhea stool samples were positive for HBoV by PCR. Rotavirus was detected in 84 (11.9%) samples, adenovirus in 34 (4.8%) samples, norovirus in 24 (3.4%) samples, and astrovirus in 2 (0.3%) samples. Enteropathogenic bacteria were found in 57 (8.1%) samples (*10**,**11*). The frequency of enteric pathogens identified in epidemiologic studies is variable (45%–54%) and dependent on several parameters such as country and type of method used for diagnosis (*12**–**14*). In our study, a potential pathogen was found in 215 (30.5%) samples (including HBoV-positive samples). No bacterial or virus pathogen was found in 499 (69.5%) samples. Samples were not tested for intestinal parasites, which in general account for ≈11% of the diarrhea etiology in developing countries (*12**,**14*).

There was no obvious temporal clustering of the HBoV-positive patients. The median age of HBoV-infected children was 1.9 years; 11 children (78.6%) were <2 years of age, 1 child was 35 months of age, 1 child was 11 years of age, and 1 child was 15 years of age. A total of 57% were boys and 43% were girls. Three patients were coinfected with other enteric viruses (1 with adenovirus, 1 with rotavirus, and 1 with norovirus). All HBoV-positive patients had diarrhea but none reported concomitant respiratory symptoms. Fever was reported in 2 patients, vomiting in 1, and bloody diarrhea in 2. One hospitalized boy (the oldest study participant) was reported to be positive for HIV and cytomegalovirus, and 1 hospitalized girl was undergoing dialysis. Ten (71.2%) HBoV-positive children were hospitalized because of diarrhea; 3 were outpatients (2 from walk-in clinics and 1 from an emergency department).

A semiquantitative PCR of HBoV in stool specimens was performed by using dilutions of DNA extracted from stool samples. We detected DNA up to a dilution of 10^–3^ in 3 samples. In the remaining samples, DNA was detected only in undiluted samples. Sequence analysis showed high nucleotide similarity between Brazilian samples and the Chinese respiratory HBoV WLL-3 strain (GenBank accession no. EF584447) from the People’s Republic of China (91.8%–99.6%) and among the Brazilian samples (96.4%–100%) (Figure). We could not compare our enteric strains with a Spanish enteric strain (*8*) because we sequenced a different portion of the virus genome. Strain MC-8 showed the lowest homology with the WLL-3 strain (91.8%) and with the other Brazilian strains (96.4%). We are conducting additional sequencing to characterize the complete genome of this strain to confirm that it represents a new variant of the virus.

**Figure:**
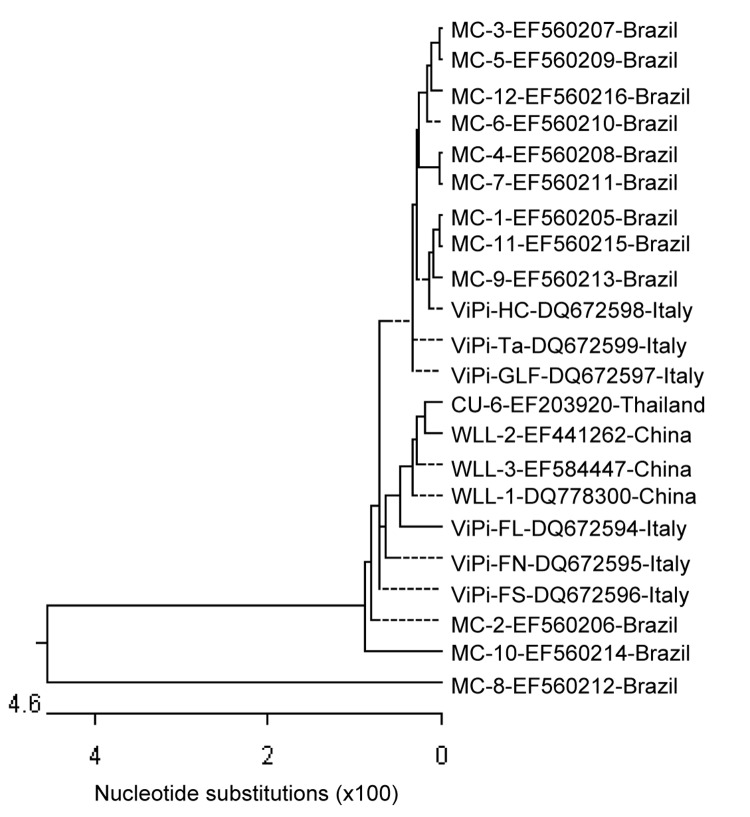
Phylogenetic analysis of nucleotide sequences of the nonstructural protein 1 gene of enteric strains of human bocavirus from Brazil. The dendrogram was constructed with the Clustal W algorithm of the MegAlign program in the Lasegene software package (DNASTAR, Madison, WI, USA). The length of each pair of branches represents the distance between sequence pairs. Dashed lines on a phenogram indicate a negative branch length. GenBank accession nos. are shown with strains.

## Conclusions

HBoV has been isolated from respiratory specimens from patients with acute respiratory illness, and increasing evidence suggests a causal relationship with this disease (*3**–**8*). The presence of HBoV in the human gastrointestinal tract was demonstrated by Vicente et al. (*8*), as well as in our study. In the first study, virus was isolated from feces of children with gastroenteritis with or without symptoms of respiratory infection. Coinfection with other intestinal pathogens was found in 28 (58.3%) of 48 HBoV-positve samples. In our study, none of the HBoV-positive patients reported respiratory symptoms. Coinfection with other enteric viruses was found in 3 (21.4%) of 14 HBoV-positive samples. High titers of DNA in some specimens suggest that the virus replicates in the human gut. However, additional studies that include control groups are needed to demonstrate an association between HBoV infection and gastroenteritis.

## References

[1] Allander T, Tammi MT, Eriksson M, Bjerkner A, Tiveljung-Lindell A, Andersson B. Cloning of a human parvovirus by molecular screening of respiratory tract samples. Proc Natl Acad Sci U S A. 2005;102:12891–6. 10.1073/pnas.0504666102 16118271 PMC1200281

[2] Berns K, Parish CR. 2007. Parvovirus. In: Knipes DM, Howley PM, Griffin DE, Lamb RA, Martin MA, Roizman B, et al. editors. Fields virology. 4th ed. Philadelphia: Wolkers Kluwer/Lippincott, Williams and Wilkins; 2007. p. 2437–77.

[3] Bastien N, Brandt K, Dust K, Ward D, Li Y. Human bocavirus infection, Canada. Emerg Infect Dis. 2006;12:848–50. 16704852 10.3201/eid1205.051424 PMC3374420

[4] Fry AM, Lu X, Chittaganpitch M, Peret T, Fischer J, Dowell SF, Human bocavirus: a novel parvovirus epidemiologically associated with pneumonia requiring hospitalization in Thailand. J Infect Dis. 2007;195:1038–45. 10.1086/512163 17330795 PMC7109861

[5] Kesebir D, Vazquez M, Weibel C, Shapiro ED, Ferguson D, Landry ML, Human bocavirus infection in young children in the United States: molecular epidemiological profile and clinical characteristics of a newly emerging respiratory virus. J Infect Dis. 2006;194:1276–82. 10.1086/508213 17041854 PMC7204143

[6] Simon A, Groneck P, Kupfer B, Kaiser R, Plum G, Tillmann RL, Detection of bocavirus DNA in nasopharyngeal aspirates of child with bronchiolotis. J Infect. 2006;54:e125–7. 10.1016/j.jinf.2006.08.001 16968654 PMC7172159

[7] Sloots TP, McErlean P, Speicher DJ, Arden K, Nissen MD, Mackay IA. Evidence of human coranaivrus HKU1 and human bocavirus in Australian children. J Clin Virol. 2006;35:99–102. 10.1016/j.jcv.2005.09.008 16257260 PMC7108338

[8] Vicente D, Cilla G, Montes M, Pérez-Yarza EG, Pérez-Trallero E. Human bocavirus, a respiratory and enteric virus. Emerg Infect Dis. 2007;13:636–7. 10.3201/eid1304.061501 17553287 PMC2725986

[9] Murphy FA, Gibbs EP, Horzinek MC, Studdert MJ, eds. Veterinary virology. 3rd ed. San Diego: Academic Press, 1999;343–56.

[10] Soares CC, Santos N, Beard RS, Albuquerque MCM, Maranhão AG, Rocha LN, Norovirus detection and genotyping for children with gastroenteritis, Brazil. Emerg Infect Dis. 2007;13:1244–6. 10.3201/eid1308.070300 17953103 PMC2828093

[11] Volotão EM, Soares CC, Maranhão AG, Rocha LN, Hoshino Y, Santos N. Rotavirus surveillance in the city of Rio de Janeiro–Brazil during 2000–2004: detection of unusual strains with G8P[4] or G10P[9] specificities. J Med Virol. 2006;78:263–72. 10.1002/jmv.20535 16372291

[12] El-Mohamady H, Abdel-Messiha IA, Youssef FG, Saidc M, Faragc H, Shaheena HI, Enteric pathogens associated with diarrhea in children in Fayoum, Egypt. Diagn Microbiol Infect Dis. 2006;56:1–5. 10.1016/j.diagmicrobio.2006.02.007 16675181

[13] Olesen B, Neimann J, Böttiger B, Ethelberg S, Schiellerup P, Jensen C, Etiology of diarrhea in young children in Denmark: a case-control study. J Clin Microbiol. 2005;43:3636–41. 10.1128/JCM.43.8.3636-3641.2005 16081890 PMC1234006

[14] Orlandi PP, Magalhães GF, Matos NB, Silva T, Penati M, Nogueira PA, Etiology of diarrheal infections in children of Porto Velho (Rondonia, Western Amazon region, Brazil). Braz J Med Biol Res. 2006;39:507–17. 10.1590/S0100-879X2006000400011 16612474

